# Discount rate heterogeneity among older households: a puzzle?

**DOI:** 10.1007/s00148-016-0623-y

**Published:** 2016-11-23

**Authors:** Antoine Bozio, Guy Laroque, Cormac O’Dea

**Affiliations:** 1 Paris School of Economics (PSE), Paris, France; 2 Institute for Fiscal Studies (IFS), London, UK; 3 University College London (UCL), London, UK

**Keywords:** Time preference, Discount rate, Consumption, D12, D31, D91, E21

## Abstract

We put forward a method for estimating discount rates using wealth and income data. We build consumption from these data using the budget constraint. Consumption transitions yield discount rates by household groups. Applying this technique to a sample of older households, we find a similar distribution to those previously estimated using field data, though with a much lower mean than those found using experiments. Surprisingly, among this older population, patience is negatively correlated with education and numeracy. This goes against the positive correlation found for younger populations in experiments and some field studies. We discuss potential explanations for this result.

## Introduction

In many situations, individuals make decisions that involve a comparison of present and future circumstances. They must decide how much to invest in education, how much to save for retirement, how much to invest in health, etc. In each case, these decisions are based on some assessment of the potential welfare at different periods under different scenarios. Following Samuelson (1937), economists have largely adopted a discounted-utility model which assumes that preferences over time can be condensed into one major parameter, the geometric discount rate (see Frederick et al. 2002 for a critical review and Hall 2010 for a review of recent research developed using this approach).

To estimate discount rates, both field data and experiments are found in the literature. Experimental studies are by far the most numerous. Among the 42 studies surveyed by Frederick et al. (2002), 34 use experimental methods. A typical approach is for individuals to be offered a menu of (real or hypothetical) choices between a quantity of money now and a different quantity of money at some point in the future. Respondents’ choices are used to estimate a discount rate.

Our paper fits into a much smaller literature that estimates discount rates using field data on aspects of behaviour and a lifecycle model of consumption and saving. A typical way to estimate preference parameters in such models, though not the one that we will take, has been to solve numerically the intertemporal optimisation problem that the agents in a particular population are assumed to face. Estimates of parameters such as the discount rate are chosen such that the model’s predictions are close, in some metric and according to some data, to those seen in reality. Such studies vary in the extent to which heterogeneity in the discount factor is admitted into the model. Some papers assume homogenous discounting behaviour, like French (2005) and Edwards (2013) where discounting is exponential and Laibson et al. (2007) where discounting is quasi-hyperbolic. More flexibility was allowed by Attanasio et al. (1999) who estimate a version of the lifecycle model where the discount rate varies stochastically with the composition of the household while even more is allowed by Samwick (1998) and Gustman and Steinmeier (2005) who estimate a different discount rate for every household.

These papers fully specify a lifecycle model and solve it. The method we employ does not do this but rather uses the first-order condition to that solution—the Euler equation. We first generate longitudinal observations on consumption using a procedure introduced by Ziliak (1998) and Browning and Leth-Petersen (2003). This involves calculating consumption using comprehensive and high quality data on assets and income and the intertemporal budget constraint. Our resulting distribution of consumption is shown to be remarkably similar to that derived from the UK’s household budget survey. Using the Euler equation and consumption transitions at the household level, we estimate average discount rates for groups of households. Such an approach has typically been precluded in the past by the absence of good quality panel data on consumption—a problem discussed in detail by Browning et al. (2003).

Our approach has some parallels with papers that have previously relied on the Euler equation to estimate parameters, in particular the elasticity of intertemporal substitution. Estimation in this manner was carried out by Campbell and Mankiw (1989) and Attanasio and Weber (1995) among others. For a lively criticism of this approach, see Carroll (2001) and for a defense see Attanasio and Low (2004). Our approach differs from these papers in three principal ways. First, we are able to use consumption transitions at the household level rather than relying on aggregate or cohort-level data. Second, our use of household rather than cohort level consumption data allows us to use the exact Euler equation in our estimation, rather than relying on Taylor series approximations. Third, we do not assume that the discount rate is the same for each individual in our sample, nor do we assume that it is unchanging across the lifecycle.

We apply the procedure outlined above to a representative sample of older English households using the English Longitudinal Survey of Ageing (ELSA). We show, unsurprisingly, that there is substantial heterogeneity in discounting in that population. The typical levels of discount rates that we estimate are of similar magnitude to those estimated in other papers based on the lifecycle model of consumption and saving. These rates imply substantially less discounting than is implied by the results of experimental studies.

Our most surprising result is that discount rates tend to rise with education and levels of numerical ability (i.e. those with less education and those who are less numerically able tend to be the most patient). This result is contrary to that found in the literature that measures the extent to which individuals discount future income streams (see for instance Warner and Pleeter 2001; Harrison et al. 2002; Dohmen et al. 2010). These papers differ in their empirical approach—the first uses data on the choices of departing military personnel over whether they will take their severance payment in a lump-sum or in the form of an annuity payment, while the second and third papers use laboratory experiments. The literature using field data offers less conclusive evidence. Gourinchas and Parker (2002) solve a lifecycle model and estimate discount rates by matching simulated consumption data to those observed in the data at different ages and, similar to us, finds that people with more education are less patient. On the contrary, Cagetti (2003), implementing a broadly similar procedure as that used by Gourinchas and Parker, but using data on assets instead of consumption, finds evidence suggesting the more educated are more patient. This is also found by Lawrance (1991), using an approach based on a log-linearised Euler equation and data on transitions in food expenditure.

We discuss our somewhat puzzling result below and raise the possibility that prior evidence, driven largely by choices individuals made at younger ages is not applicable to the discounting between periods at older ages.

The rest of this paper is structured as follows. Our empirical approach is outlined in Section 2. In Section 3, we describe the data and explain how we calculate consumption from assets and income. Results are presented in Section 4 and discussed in Section 5. Section 6 concludes.

## Theory and empirical approach

In our estimation of discount rates, we start from a standard life-cycle model in which each household (as a collective unit) maximises expected discounted utility by choosing their consumption and their holdings of each of *J* different asset or debt instruments each period. In period *t*, household *i* faces the following optimisation problem:

$$\max\limits_{\{X^{j}_{is}, c_{is}\}_{s=t}^{T}}\qquad u(c_{it}) + \sum\limits_{\tau = t + 1}^{T} \left( \prod\limits_{s=1}^{\tau - t} \frac{1}{1+\rho_{i(t+s)}}\right) E \left[ u(c_{i\tau})\right] $$

subject to the constraints

(i) 1 $$ p_{\tau} c_{i\tau} + {\sum}_{j} p^{j}_{(\tau+1)} X^{j}_{i(\tau+1)} = e_{i\tau} + d_{i\tau}+ {\sum}_{j} r^{j}_{\tau} p^{j}_{\tau} X^{j}_{i\tau} + {\sum}_{j} p^{j}_{\tau} X^{j}_{i\tau} \;\; \; \forall \;\tau  $$
(ii) 2 $$ X^{j}_{i(\tau+1)} \ge b^{j}_{i(\tau+1)} \; \;\; \forall \; \tau, j  $$

where *ρ*_*it*_ is the discount rate for household *i* between period *t* and *t*+1. Equation 1 is the budget constraint at date *τ* and Eq. 2 represents a borrowing constraint for asset *j*: *b* ^*j*^ is the minimum level of that asset that must be held. This will be negative for debt instruments that households have access to and zero for non-debt instruments.^1^ The other quantities in the model are consumption (*c*_*t*_), holdings of each of *J* assets (${X_{t}^{j}}$) which are negative in the case of debts, the nominal income yield of asset $j\,({r^{j}_{t}})$, the price of asset $j ({p^{j}_{t}})$, labour income (*e*_*t*_), income from transfers (*d*_*t*_) and the price of consumption (*p*_*t*_). We make the standard assumption that the instantaneous utility function *u*(.) is invariant over time.

An Euler equation (first-order condition) is satisfied for every asset that households can potentially hold (see for example Campbell 2000). That is, for each asset *j*, and for each pair of consecutive periods *t* and *t*+1, the following inequality holds:

3 $$ \frac{\mathrm{d} u(c_{it})}{\mathrm{d} c_{it}} \ge \frac{1}{(1 + \rho_{i(t+1)})} E \left[ \left( 1 + r^{j}_{t+1}\right) \frac{p_{t}}{p_{(t+1)}} \frac{\mathrm{d} u(c_{i(t+1)})}{\mathrm{d} c_{i(t+1)}}\right]  $$

The Euler equation holds at equality for household *i* as long as the sales of asset *j* are not constrained (i.e. as long as $X^{j}_{it+1} > b^{j}_{it+1}$). In particular, the consumption of households who hold positive cash balances satisfies (where 0 indexes cash):

4 $$ \frac{\mathrm{d} u(c_{it})}{\mathrm{d} c_{it}} = \frac{1}{(1 + \rho_{i(t+1)})} E \left[ \left( 1 + r^{0}_{t+1}\right) \frac{p_{t}}{p_{(t+1)}} \frac{\mathrm{d} u(c_{i(t+1)})}{\mathrm{d} c_{i(t+1)}}\right]  $$

All features of the model other than the instantaneous utility function are allowed to vary freely over time—in particular, we do not assume that the discount rate is time-invariant.

We specify the utility function as taking the familiar isoelastic form:

5 $$ u (c) = \frac{c^{1-\gamma }}{1- \gamma}  $$

where *γ* is the coefficient of relative risk aversion. Assuming that constraints on cash holdings do not bind, the discount rate is given by (suppressing *i* subscripts):

6 $$ \rho_{t+1} = E \left[ \left( 1+r^{0}_{t+1}\right) \frac{p_{t}}{p_{t+1}} \left( \frac{c_{t}}{c_{t+1}}\right)^{\gamma} \right] - 1.  $$

This equation forms the basis for our empirical approach. We group households according to particular characteristics (such as their education level, numerical ability, age and marital status) and estimate the expectation in the equation above by using the sample average of the quantity in square brackets among households of a particular group. In using the sample average to estimate the expectation term, we need to assume that there are no differential shocks across households that lead to systematic differences in the term $\frac {p_{t}}{p_{t+1}} \left (\frac {c_{t}}{c_{t+1}}\right )^{\gamma }$.

It is worth pointing out how the change in consumption (the central observable quantity that enters the Euler equation) identifies the discount rate. The faster is consumption growth, all else being equal, the lower is the discount rate (that is, the more patient is the individual concerned). Households who are patient tend to forsake current consumption for future consumption—and therefore exhibit consumption growth. The converse is also true. Those who are impatient tend to prefer current consumption to future consumption. They therefore have lower (or even negative) consumption growth.

**Fig. 1:**
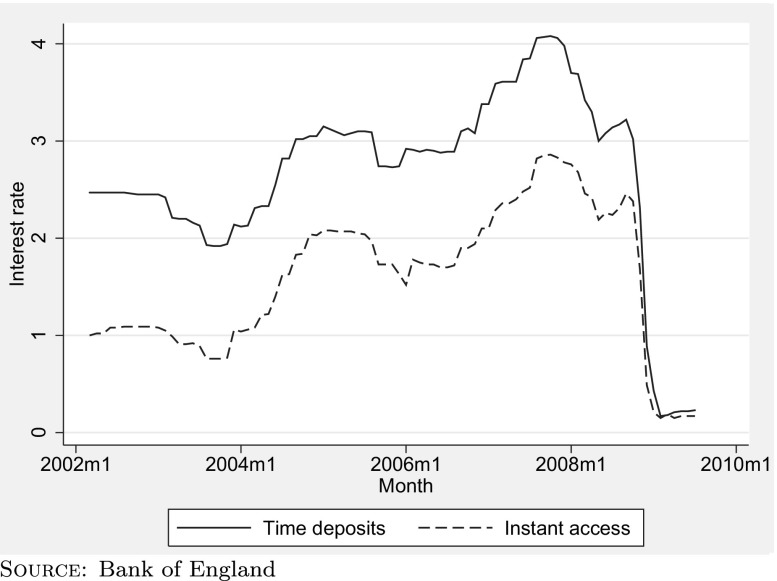
Nominal pre-tax rate of return on cash in the UK – 2002 to 2009

To bring Eq. 6 to the data, we need to specify an interest rate on cash and a coefficient of relative risk aversion. We now discuss each of these in turn. Figure 1 shows the nominal pre-tax rate of return on two types of cash assets in the UK between 2002 and 2009—instant access savings and time deposits. Until the large fall in the last quarter of 2008, interest rates were relatively stable, moving within a range of approximately a percentage point. In our estimation, we use a nominal pre-tax interest rate of 3 %—approximately the average rate of return on time deposits over the period. Our headline results refer to the consumption changes over the period 2004 to 2006 and will therefore be unaffected by the large fall in interest rates in 2008. With some exceptions, interest is taxable in the UK and we convert this pre-tax interest rate to a post-tax interest rate using the marginal rate of tax faced by each household in a particular year. For couples whose members face different marginal tax rates, we use the lower of the two rates, on the basis that efficient tax-planning in most cases will allow the couple to pay that lower rate of tax on their asset income.

We cannot identify the coefficient of relative risk aversion (*γ* in Eq. 6) and we assume that is does not vary across individuals. The assumption of a coefficient of relative risk aversion that does not vary across households is a strong one (Outreville 2015 surveys the empirical literature which has shown differences in risk aversion exist by education group). However, our data does not contain sufficient individual variation in interest rates to warrant identification. We set *γ* equal to 1.25. This is consistent with the range of elasticities of intertemporal substitution estimated by Attanasio and Weber (1993) on UK data and very close to that obtained by Gustman and Steinmeier (2005). We have generated results assuming alternative values of *γ*. While the mean discount rates are sensitive to these values, the ranking of households’ discount rates is the same for all positive choices of *γ* that are constant across households.

Finally, it is worth making explicit two restrictions implicit in the use of Eq. 6. These relate to liquidity constraints and changes in the utility function due to changing household composition or changing labour supply.

First, recall that the Euler Eq. 6 only holds at equality when individuals are not liquidity constrained. If we use Eq. 6 to estimate the discount rate for a group containing liquidity constrained individuals, the estimate will be biased downwards. Concerns about the presence of liquidity constraints in our case are mitigated by the fact that we work on a population over the age of 50, at a point in the lifecycle where most have accumulated some liquid wealth—almost 95 % of our sample have positive gross liquid asset holdings. As a check that our results are not being driven by liquidity constraints, we have confirmed that there are no substantial changes in focusing only on those with liquid assets above a certain minimum level (see Section 5).

Second, when individuals leave or join a household between periods, the assumption of a constant instantaneous utility function at dates *t* and *t*+1 does not make sense; we therefore do not include households whose composition changes between waves of data. Further, as evidence points to changes in consumption patterns around retirement (Banks et al. 1998; Wakabayashi 2008), we exclude from our sample households where some member left the labour market during the period covered by our data.

The previous two paragraphs have outlined two exclusions from our estimating sample. Some further exclusions are necessary due to the fact that we are not able to calculate consumption satisfactorily for every household in our sample. The extent of these exclusions is outlined in the A. To deal with the fact that those omitted are unlikely to be a random sub-sample of our overall sample, we generate weights representing the probability of each household being included in our sample and, in our results we attach a weight to each household of the inverse of this probability. These probabilities are estimated as functions of marital status, education, age, income quintile and wealth quintile. Our results will, therefore, be representative of the entire population aged 50 and over if the selection into our sample can be adequately modelled as a function of these characteristics.

## Data

Our data come from the English Longitudinal Study of Ageing (ELSA). ELSA is a panel survey that is representative of the English population aged 50 and over. It started in 2002, and individuals have been re-interviewed every 2 years since then—our main results use data from the first three waves. The purpose and form of the survey is similar to the Health and Retirement Study (HRS) in the US and the Survey of Health, Ageing and Retirement in Europe (SHARE) in 20 European countries. The first wave was conducted between April 2002 and March 2003 and sampled 12,099 individuals (of whom 11,391 were core sample members; the remainder was individuals aged under 50 who were the partners of core sample members). There are 7894 benefit units (i.e. a single person or couple along with any dependent children) where each member of the couple is a sample member. Our sample is drawn from these benefit units.

While ELSA contains questions on some components of expenditure (food, domestic fuel and clothing) it does not, unfortunately, contain data on total expenditure, which, approximating consumption, is needed to estimate the discount rate. In fact, there is no nationally representative longitudinal survey that collects total expenditure in the UK and such data is rare internationally.^2^ This lack of comprehensive longitudinal data on expenditure has proved something of an obstacle to bringing Euler equations to data. The literature has either relied on aggregate data, or, following Browning et al. (1985), has used repeated cross-sections to form a quasi-panel of birth cohort-level average expenditure. An alternative approach was suggested by Skinner (1987) and refined by Blundell et al. (2008). It involves estimating the relationship between food (and possibly other items of) expenditure and total expenditure using a household budget survey. As general-purpose panel surveys often contain data on food expenditure, this estimated relationship can be used to impute total expenditure.

We proceed in a different manner: following Ziliak (1998) and Browning and Leth-Petersen (2003), we use the rich data on income and assets that is contained in ELSA to back out expenditure from the intertemporal budget constraint. In all the following, we will equate total expenditure (excluding mortgage repayments) with consumption. The rest of this section summarises this procedure—further details are given in the A—and shows a close correspondence between features of the resulting distribution of consumption with those that are obtained using the UK’s Household Budget Survey.

### Calculating consumption using longitudinal data on assets and income

We use longitudinal data on assets and income along with the budget constraint to calculate consumption between two waves. Equation 1 can be re-arranged to get the value of consumption in period *t* as follows:

7 $$ p_{t} c_{t} = e_{t} + d_{t} + \sum\limits_{j} {r^{j}_{t}} {p^{j}_{t}} {X^{j}_{t}} + \sum\limits_{j} \left( {p^{j}_{t}} {X^{j}_{t}} - p^{j}_{t+1} X^{j}_{t+1}\right)  $$

The timing convention and how it relates to the data deserves some discussion. In ELSA, interviews take place approximately every two years. So to be precise,

- Flow variables representing consumption *c*_*t*_, non-capital income *e*_*t*_, transfers *d*_*t*_ and the asset yield, including any capital gain or loss, ${r^{j}_{t}}$, are measured over the entire two year period;
- Stock variables ${X^{j}_{t}}$ represent holdings of assets at the beginning of the period (i.e. at the time of the first interview); $X^{j}_{t+1}$ represents asset holdings at the beginning of period *t*+1 or equivalently at the end of period *t* (i.e. at the time of the next interview);
- Asset prices ${p^{j}_{t}}$ and $p^{j}_{t+1}$ represent asset prices at the time of the first and second interview;
- The overall price level *p*_*t*_ represents the average price level in the period between the two interviews.

Equation 7 is the equation that we use to calculate consumption between two waves of the survey. Having calculated consumption in this manner, we make one further adjustment and subtract mortgage repayments (both capital components and interest) from the resulting quantity. While these represent cash expenditure on housing, they are not generally indicative of consumption of the flow of housing services.

Equation 7 can be rewritten as:

8 $$ p_{t} c_{t} = e_{t} + d_{t} + \sum\limits_{j} \left( {q^{j}_{t}} + \frac{ p^{j}_{t+1} - {p^{j}_{t}}}{{p^{j}_{t}}}\right) {p^{j}_{t}} {X^{j}_{t}} + \sum\limits_{j} \left( {p^{j}_{t}} {X^{j}_{t}} - p^{j}_{t+1} X^{j}_{t+1}\right)  $$

where the rate of return on asset *j*, ${r^{j}_{t}}$ has been written as the sum of the income yield ${q^{j}_{t}}$ and the capital gain $\frac { p^{j}_{t+1} - {p^{j}_{t}}}{ {p^{j}_{t}}}$ earned during period *t*.

Some, but not all of the quantities on the right-hand-side of Eq. 8 can be directly read from the ELSA data. In each wave the value of each asset held (*p* ^*j*^ *X* ^*j*^) is recorded, as are non-capital income (*e*), capital income (*q* ^*j*^ *p* ^*j*^ *X* ^*j*^) and lump-sum transfers (*d*) in the period prior to the interview (where ‘period’ in the case of most forms of income and transfers represents 12 months). ELSA respondents are asked for details on 16 different financial asset and (non-mortgage) debt instruments.^3^ These include various type of savings products, bond holdings and equity holdings. See Section A in the Appendix for more detail and Table 10 in that section for summary statistics on holdings in each asset.

Our data does not record capital gains on assets held between the two waves ($\frac {p^{j}_{t+1} - {p^{j}_{t}}}{{p^{j}_{t}}}$), nor does it contain data on income and transfers for a period of approximately one year (recall that ELSA sample members are surveyed approximately every two years) - two objects that appear in Eq. 8. The majority of assets held by the population in our sample are in safe forms—so there is no capital gain to be considered for these assets. For equity holdings, we assume a capital gain (or loss) in line with the change in the FTSE index between the two interview dates. Estimating income in the missing year is facilitated by exploiting the longitudinal aspect of the survey data—we interpolate linearly between income in year *y* and income in year *y*+2 to obtain income in year *y*+1. Finally, we assume that there are no lump-sum transfers in the missing year and we exclude from our sample those households where it is likely that some member received a lump-sum transfer (due to retirement, redundancy or the death of a spouse or parent). We give further details about all of these assumptions and their implications in the A.

### Comparing consumption in ELSA and in the EFS

In this section, we compare the distribution of consumption estimated in the manner described above with that estimated using the Expenditure and Food Survey (EFS).^4^ The EFS is the UK’s household budget survey, and is used to calculate the commodity-weights of the UK’s inflation indices (the Consumer Prices Index and the Retail Prices Index). The data is collected annually, throughout the year and the survey is designed to be nationally-representative. Respondents are asked to record all purchases over a 2-week period in a diary and also to complete a questionnaire that seeks information on infrequently-purchased items. The combination of the diary and the questionnaire allows a comprehensive measure of consumption to be calculated.

**Fig. 2:**
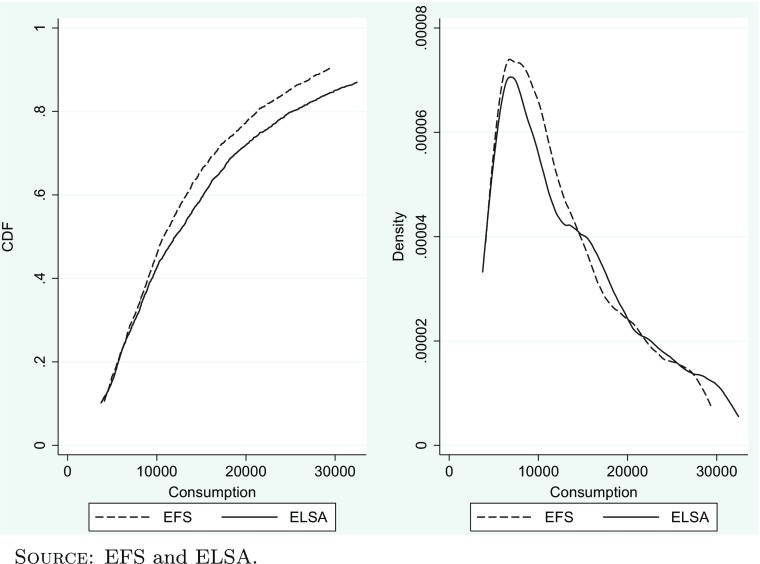
CDF and PDF of consumption in EFS and ELSA—2003

Figure 2 shows the cumulative distribution function and the probability density function of total consumption in both surveys. The data shown is for calendar year 2003 for the EFS and for (annualised) calculated consumption between the surveys in 2002/03 and 2004/05 for ELSA. The EFS functions are estimated using only those households where the head is aged over 50 so that both samples are drawn from populations with the same age profile. Both distributions are shown net of mortgage repayments. As mentioned at the end of Section 1 and discussed in the A, to compute the distribution of consumption in ELSA, we weight each observation by the inverse of the probability of being able to calculate consumption. In both surveys, we trim the most extreme values—showing the middle 80 % of the distribution.

Figure 2 shows that there is a close correspondence between the distributions in both shape and location. The correspondence is closest at the bottom of the distribution (i.e. up to annual consumption of £10,000). At this point, the distributions diverge somewhat—with the distribution of consumption in ELSA lying to the right of that in the EFS. This divergence, which represents a tendency for consumption to be greater in ELSA than the EFS in the upper half of the distribution, is consistent with the fact that consumption in the EFS (grossed up to national levels) is known to under-record the level of consumption calculated as part of the National Accounts with the degree of under-reporting thought to be greater for those who have higher levels of consumption (see Brewer and O’Dea 2012).

### Summary statistics

We noted at end of the last section (and give more detail in Appendix A) that some households are omitted from the sample (for example, because the data on their asset holdings had to be imputed and therefore we are not confident in the quality of our consumption data). Table 1 gives summary statistics, both for the full ELSA sample and our sample. Panel (A) gives proportions in categories of age, education, numerical ability and marital status: these are the variables which we use to group households in estimating discount rates (6).^5^ Panel (B) gives means and selected percentiles of the distribution of annual income, net liquid wealth and net housing wealth. Comparisons between the two sets of statistics shows that there is a close correspondence between the characteristics of our sample and the full ELSA sample—with the exception that our sample under-represents those with the highest liquid wealth holdings.

**Table 1:** Summary statistics

(A)			(B)		
	All	Our		All	Our
	Households	sample		Households	sample
Age groups (props.)			Annual income (£1000s)		
50–59	34.45	34.13	Mean	17.01	17.14
60–69	26.78	25.29	p10	5.08	5.14
70–79	23.84	23.95	p25	7.58	7.70
80 +	14.93	16.63	p50	12.69	13.30
Total	100.00	100.00	p75	21.10	21.58
			p90	32.36	31.09
Education (props.)			Net liquid wealth (£1000s)		
Low	54.70	54.65	Mean	73.28	45.00
Middle	28.57	28.24	p10	0.00	0.00
High	16.66	17.10	p25	1.61	2.00
Total	100.00	100.00	p50	13.50	11.52
			p75	61.55	51.60
			p90	169.00	121.36
Numerical Ability (props.)			Net housing wealth (£1000s)		
1(Lowest)	11.89	9.73	Mean	114.11	117.03
2	41.22	40.97	p10	0.00	0.00
3	29.95	32.53	p25	0.00	20.00
4(Highest)	15.47	16.07	p50	90.00	95.00
Missing	1.47	0.70	p75	161.00	175.00
Total	100.00	100.00	p90	250.00	260.00
Marital status (props.)			Sample size		
Single	7.19	8.42		7,894	1,504
Marr/cohab	52.08	54.32			
Widowed	24.12	26.23			
Sep/Div	10.48	11.03			
Other	6.15	0.00			

## Results

We first summarise the distribution of the quantities represented by the right-hand side of Eq. 6:

9 $$ \left[(1+r^{0}_{t+1})\frac{p_{t}}{p_{t+1}}\left( \frac{c_{t}}{c_{t+1}}\right)^{\gamma}\right] - 1 $$

This quantity would be equal to the discount rate if *c*_*t*+1_ and *p*_*t*+1_ were perfectly forecasted by households at date *t*. Our presentation of the distribution of this quantity which we refer to below as the ‘ex-post’ discount rate is a useful preliminary step.

**Fig. 3:**
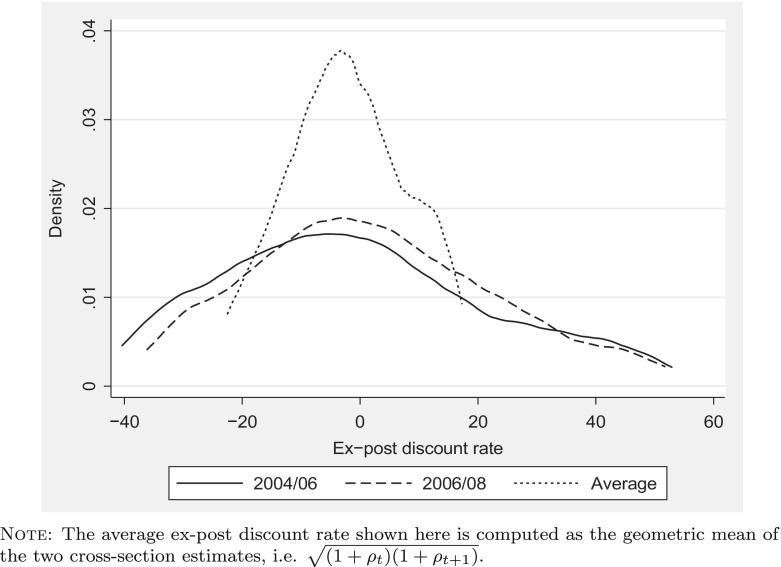
Distribution of ex-post discount rates – 2004/06, 2006/08 and average

Our use of four waves of ELSA data gives us up to three observations on consumption for each household and therefore up to two observations on the ex-post discount rate. Figure 3 shows two distributions of the ex-post discount rates (trimming the bottom 10 % and top 10 % of the sample). The median discount rate is approximately −3 % in the earlier period and is 0 % in the later period. These median ex-post discount rates are low relative to estimates of the discount rate found in the literature that estimate such rates using field data and very low relative to those found in the experimental literature.

Figure 3 shows substantial heterogeneity around these medians. This depicts the distribution of the discount rate, perturbed by two phenomena. First, realisations of stochastic variables will differ from their expectations. Second, our consumption data is likely to include some measurement error. Figure 3 also shows the distribution of the geometric mean of our two successive observations on the discount rate. The variance of this distribution is substantially smaller than the variance of either cross-sectional distribution. This could be due to some combination of averaging over time of the discount rate for each family and to a diminished effect of measurement error once we take a time average.

We are aware of three papers that, using field data and the lifecycle model, have estimated the entire distribution of discount rates: Alan and Browning (2010), Samwick (1998) and Gustman and Steinmeier (2005) (hereafter GS). In Table 2, we compare our three distributions of the ex-post discount rate (the two cross-sectional distributions, and the distribution of their geometric average) to those found in the last two of these using a breakdown reported in GS.^6^ It is important to note, though, that we would not necessarily expect a close correspondence between our results and theirs as the populations on which the estimates are based are very different. Our results are for English households containing an individual aged over 50, while the results of both Samwick and GS are estimated on samples of US working-age adults.

**Table 2:** Comparison of our results with those of Samwick (1998) and Gustman and Steinmeier (2005)

Discount rate	Samwick	GS	Ours 04–06	Ours 06–08	Ours Ave
<5 %	38 %	40 %	60 %	56 %	67 %
5–10 %	25 %	21 %	5 %	6 %	9 %
10–15 %	10 %	6 %	5 %	6 %	7 %
>15 %	25 %	33 %	30 %	32 %	17 %

Notes: These groups are those reported in Gustman and Steinmeier’s Table 2. That table does not show the proportion with negative estimated discount rates. Our three estimated distributions have proportions with negative estimated discount rates of 53, 48 and 56 %, respectively

The most striking difference between our results and those of Samwick and GS is the substantial number of households that we find with ex-post discount rates of less than 5 %. We find approximately 60 % here in this portion of the distribution compared to approximately 40 % in the distribution of discount rates in the two papers based on the US all-age population. We find a larger share of households with negative ex-post discount rates (approximately 50 % of the sample). This compares to approximately 10 % of those in Samwick’s sample (see his Fig. 3; note that GS do not report the proportion with negative discount rates).

In our two cross-sectional distributions, we find a similar share of households in the right tail of the distribution (those with a discount rate greater than 15 %) as do Samwick and GS, and find less mass in the region of 5 to 15 %. On our average measure, the mass in the left tail of our distribution increases, largely at the expense, relative to either cross-sectional distribution, of that in the right tail.

The models of both Samwick and GS assume a discount rate for a particular household that does not change over time. If discount rates do vary over time, their estimates represent some average of the lifetime sequence of discount rates. Therefore, the large mass that we find in the left tail of the distribution could be reconciled with the estimates of Samwick and GS if households have higher discount rates at younger ages than at older ages (at which point they enter our population of interest).

Table 3 gives estimates of the median ex-post discount rate ($\hat {\rho }$) and associated standard errors of the medians (*σ*), for groups defined according to age, marital status, education, numerical ability (we discuss how these last two variables are constructed in A). No clear relationship with age is evident^7^ while the evidence is suggestive that, if anything, those who are widowed or divorced are more patient in this period than those who are single and never married and those who are married. Surprisingly, we find that less educated families and families with lower levels of numerical ability tend to be more patient than those with more education and greater levels of numerical ability respectively (though the differences are less pronounced in the latter case).

**Table 3:** Median ex-post discount rate by household characteristics

Age	$\hat {\rho }$	*σ*	Marital status	$\hat {\rho }$	*σ*	Education	$\hat {\rho }$	*σ*	Numerical ability	$\hat {\rho }$	*σ*
50–59	−2.2	2.4	Single	0.1	5.3	Low	−3.2	1.0	1 (Low)	−2.9	2.0
60–69	−4.6	1.9	Married	−2.1	2.1	Mid.	−1.8	2.1	2	−3.2	1.1
70–79	−2.5	1.4	Widowed	−3.1	1.7	High	6.5	5.6	3	−0.8	2.7
80 +	0.5	2.5	Sep./Div.	−4.8	2.1				4 (High)	−1.3	4.0
All	−2.3	1.0	All	−2.3	1.0	All	−2.3	1.0	All	−2.3	1.0

Notes: The number of households in each of the age groups are 350, 432, 492 and 229. The number of households in each of the marital status groups are 133, 619, 510 and 241. The number of households in each of the education groups are 942, 396 and 165. The number of households in each of the four numeracy groups are 189, 699, 431 and 174. The median in the ‘All’ row differs slightly between columns as the number of households in each differs. In calculating the ‘All’ group median, we exclude those with missing values of the covariate in question

We use a grouping estimator to estimate the average ex-ante discount rate for groups defined by age, marital status, levels of education and numerical ability. The estimator is based on Eq. 6. It estimates the expectation in that equation by its sample analogue for a particular group and weights the results to account for possibly non-random selection into our sample. For each group, we trim the sample, removing those in the first and tenth decile of consumption growth. Unlike Alan and Browning (2010), we do not explicitly account for the role of measurement error. However, our approach does not involve assuming (as does the vast majority of work in this area) that preference parameters remain the same over the whole of the lifecycle.

Table 4 summarises these results. The results mirror those presented above in Table 3—no clear relationship with age; widows and those who are divorced appearing more patient than those who are otherwise single and those who are married; and evidence that discount rates increase with education and numerical ability.

**Table 4:** Mean ex-ante discount rate by household characteristics

Age	$\hat {\rho }$	*σ*	Marital status	$\hat {\rho }$	*σ*	Education	$\bar {\rho }$	*σ*	Numerical ability	$\bar {\rho }$	*σ*
50–59	−0.0	1.4	Single	1.2	2.2	Low	−2.6	0.7	1 (Low)	−2.9	1.4
60–69	−1.8	1.2	Married	2.0	1.2	Mid.	0.8	1.4	2	−2.4	0.8
70–79	−0.7	1.1	Widowed	−3.2	1.0	High	6.7	3.0	3	1.6	1.5
80 +	−0.5	1.7	Sep/Div	−3.8	1.2				4 (High)	2.3	2.4
All	−0.9	0.6	All	−0.9	0.6	All	−0.9	0.6	All	−1.0	0.6

Notes: The sample sizes in each category are smaller here than in Tables 3 as, in each category we trim the top and bottom 10 % of values. The number of households in each of the age groups are 280, 343, 392 and 183. The number of households in each of the marital status groups are 107, 499, 408 and 193. The number of households in each of the education groups are 749, 319 and 133. The number of households in each of the four numeracy groups are 153, 561, 345 and 140. The mean in the ‘All’ row differs slightly between columns as the number of households in each differs. In calculating the ‘All’ group median, we exclude those with missing values of the covariate in question

The magnitude of the differences between the education groups is large.^8^ For example, the difference between the point estimates of the mean discount rate for the ‘low’ education group and those in the ‘high’ education group is over 9 percentage points. This means that, over this period and in this population, if those with the most education are to exhibit the same saving behaviour at the margin as those with the least, the former group will require a safe return of 9 percentage points greater than the latter group.

Table 5 investigates the joint association between average discount rates, education and numerical ability. Here, those in numeracy groups 1 and 2 are categorised as having ‘low’ numerical ability and those in numeracy groups 3 and 4 are categorised as having ‘high’ numerical ability. The ‘low’ education group is defined as before, while the ‘med./high’ education group contains the upper two categories. The gradient of the association between education and average discount rate, conditional on level of numerical ability is particularly large. Among those with low levels of numerical ability, the average discount rates of the low education group is estimated at −3.1 %, compared to 1.0 % for those in the mid./high education group. For those with more numerical ability, the differences according to education are starker—with average discount rates of −1.5 % and 4.6 % for those with less and more education, respectively.

**Table 5:** Mean ex-ante discount rate for groups defined by pairwise combinations of both education and numerical ability

	Low education	Med./High education
Low numeracy	−3.1	1.0
	(0.8)	(1.8)
High numeracy	−1.5	4.6
	(1.5)	(1.9)

Standard errors are in parentheses

Table 6 explores the robustness of our most puzzling result—the fact that estimated mean discount rates are higher for those with more education than those with less. Column 1 is associated with less trimming than in our headline results. We trim those in the bottom and top 5 % of the distribution of consumption growth instead of those in the bottom and top 10 %. Columns 2 and 3 apply successively stricter sample selection rules than are applied in our baseline sample. These are the ‘middle’ and ‘strict’ sample selection rules outlined in Appendix A. In the first two cases, the results that we previously emphasised still hold—the estimated mean discount rates are higher for those with more education than those with less. In columns 2 and 3, as the sample becomes more restricted and smaller, the gradients are less clearly monotonic and the standard errors are larger.

**Table 6:** Sensitivity of mean ex-ante discount rate for groups defined according to education

Education	(1)		(2)		(3)	
	$\bar {\rho }$	*σ*	$\bar {\rho }$	*σ*	$\bar {\rho }$	*σ*
Low	−1.0	0.9	−1.5	0.9	−0.6	1.3
Mid.	3.9	1.8	2.7	1.8	3.0	2.6
High	10.5	3.6	3.9	4.2	−0.3	6.8
All	2.1	0.8	−0.5	0.8	−0.3	1.2

The total sample size is 1355 in column (1), 776 in column (2) and 305 in column (3)

We might want to confirm that our relatively small sample, combined with our trimming of the largest 10 % increases in consumption and largest 10 % decreases in consumption does not materially affect our results. To investigate this, we solve and simulate behaviour from a simple life-cycle model. We specify a life-cycle model where agents make a consumption and saving choice every year from the age of 20 to 100. In each period until the age of 65 they receive income which follows an autoregressive process with autocorrelation of 0.95 and variance of 0.02. We specify that there are equal numbers of ten types of agents, each with a different discount rate. Discount rates range from −2.5 to 7.5 % with a mean of 2.5 %.

After solving (using a backwards recursion) for the set of consumption functions, we carry out the following procedure 499 times. First, we draw a sample of 200 agents (20 from each discount rate type). Second, we simulate their consumption behaviour using the calculated consumption functions. Third, we take one consumption transition between two sequential years for each individual. The age for the transition is chosen as a random age between 50 and 80; this age differs for each individual. We then trim the largest and smallest 10 % of consumption transitions and estimate a discount rate (just as we do in our estimation). The mean (across 499 simulations) estimated discount rate is 2.37 % with a 95 % confidence interval of [2.16 %, 2.59 %] which contains the truth. This simulation reassures us that estimation of the discount rate in sample sizes of the type that we have is possible with reasonable precision.

## A puzzling result?

Our results are puzzling in light of the studies, particularly those using experimental designs, that find that low educated individuals tend to lack patience compared to higher educated. A primary difference between this paper and most of the rest of the literature is the fact that results in the latter come from samples of individuals who tend to be much younger than ours. Work using lifecycle models (e.g. Samwick 1998; Gustman and Steinmeier 2005) typically focuses on working age individuals while the experimental literature often uses samples of students. In contrast, our sample comprises older households in England, aged 50 and above. Frederick et al. (2002) noted at that time that ‘no studies [had] been conducted to permit any conclusions about the temporal stability of time preferences’ and, while Bishai (2004) does investigate how time preference changes over the age range 14 to 37, we are not aware of any studies that look at the discounting behaviour of the oldest households. We now briefly consider some other explanation for our results.

First, consider survival probabilities. Households in the model discount the future for two reasons—first their ‘pure’ rate of time preference, and second their expectation of being alive at each period in the future. Differential survival probabilities could explain our result on education in the following manner. Suppose that all education groups had the same mean ‘pure’ discount rate, but that one group had a longer life-expectancy. This group would appear to be more patient. A negative correlation between education and life expectancy could explain, at least some of, our results. In our data, however, it is those with lower education levels that tend to have shorter life expectancies. ELSA respondents are asked the following question: ‘What are the chances that you will live to be X or more?’, where X depends on their current age. We run a simple linear regression of the responses to this question on dummies for our education groups and age dummies (the latter to account for the possibility that those in different education groups are distributed differently across ages). We find that those in the middle education consider themselves to be 3.1 percentage points more likely to live to the age referenced in the question than those in the low education group, and those in the high education group are 5.4 percentage points more likely. Differential life expectancies, therefore, would seem to work in the opposite direction from our puzzle.

Second, if it were the case that those with more education had access to higher pre-tax safe rates of return, then our assumption that everyone has the same pre-tax rate of return would generate a downward bias in the estimates of discount rates for the more educated relative to the less. That those with more education might face (through greater financial literacy) higher rates of return is plausible. This would render our result a conservative one—and the actual gap between the discount rate of those with less and more education could be greater than that which we find.

Third, liquidity constraints might be important. Recall that for any household that is liquidity constrained, the Euler equation (which forms the basis for our estimating Eq. 6), will hold with an inequality rather than an equality. Our sample is comprised of those over the age of 50 who are substantially less likely to be liquidity constrained than those earlier in their lifecycle; Table 10 in the Appendix shows that 93 % of our sample have positive holdings of gross liquid assets. To investigate a potential differential incidence of liquidity constraints between those with different levels of education and numerical ability, we show results for samples restricted to those with holdings of at least £2500, £5000 and £10,000 of gross liquid assets, respectively. Table 7 shows the results for these sub-samples for our groups defined by education. The gradient of interest—that patience is decreasing in education—is apparent in each of the sub-samples. While the populations represented by each of these sub-samples differ in important ways (because, for example, wealth is endogenous to the discount rate), we interpret these results as strongly suggestive that liquidity constraints are not driving our headline associations between discount rates and education.

**Table 7:** Mean ex-ante discount rate for groups defined according to education and level of positive gross liquid assets

Education	Level of positive liquid gross assets							
	Base		≥ 2,500		≥ 5,000		≥ 10,000	
	$\bar {\rho }$	*σ*	$\bar {\rho }$	*σ*	$\bar {\rho }$	*σ*	$\bar {\rho }$	*σ*
Low	−2.6	0.7	−5.2	1.0	−5.3	1.3	−5.2	1.8
Mid.	0.8	1.4	−0.2	1.8	−0.4	2.1	−1.9	2.4
High	6.7	3.0	8.0	3.4	8.7	3.4	6.9	3.6
All	−0.9	0.6	−2.4	0.9	−1.0	1.1	−1.2	1.4

The total sample size is 1,200 in the base category, 818 in the sample of those with over £2,500, 641 in the sample with over £5,000 and 497 in the sample with over £10,000

**Fig. 4:**
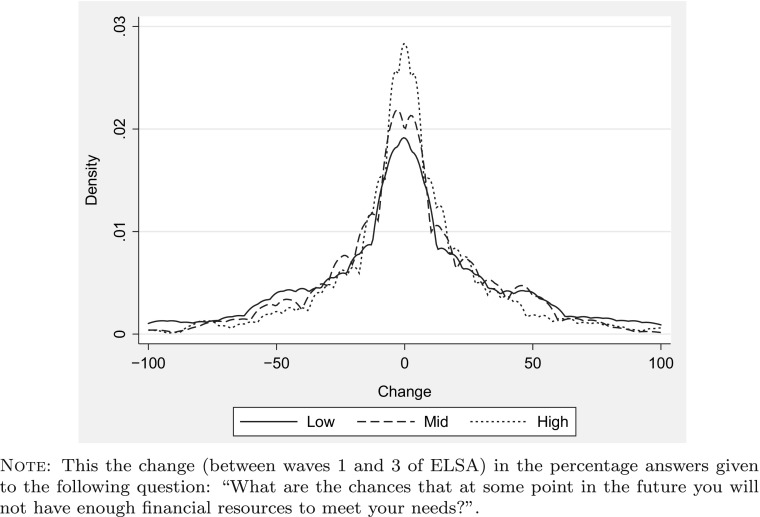
Variation in financial insecurity by education level

A fourth consideration is the possible incidence of non-insured shocks that differ systematically between the groups we examine. The effect of these will not be removed from the grouping estimator that we implement. The ELSA data allows us to indirectly assess whether these may be important. There is a question which asks ‘What are the chances that at some point in the future you will not have enough financial resources to meet your needs?’. Figure 4 shows the distribution of changes in financial insecurity reported by individuals between waves 1 and 3 for the three different education groups. There is a peak at no change for all three education groups - with no evident differences in the location between groups. Linear regressions of these changes on education dummies and dummies for numerical ability reveal no (even marginal) statistically significant differences between groups. We take this as suggestive evidence that there were not substantial differential shocks across education groups over our data period.

As a fifth check, it is useful to assess, to the extent that we can with our short panel, whether the relationship between education and the discount rate is also found using consumption transitions between time periods other than those we have focussed on above. The results described in the previous section are generated using differences in consumption between the period 2002–2004 (between waves 1 and 2 of ELSA) and the period 2004–2006 (between waves 2 and 3 of ELSA). We have also estimated discount rates using the change in consumption between this second period and 2006–2008 (the period between waves 3 and 4 of ELSA). These results are shown in Table 8, alongside our baseline results. The differences in patience between the most and least educated group are larger in the case of the latter pair of years, though there is little difference over that period between the estimated discount rates between the lower two education groups.

**Table 8:** Mean ex-ante discount rate between two different set of years

Education	2002/04–2004/06		2004/06–2006/08	
	$\bar {\rho }$	*σ*	$\bar {\rho }$	*σ*
Low	−2.6	0.7	0.7	0.7
Mid.	0.8	1.4	−0.2	1.3
High	6.7	3.0	12.4	2.5
All	−0.9	0.6	2.0	0.6

A sixth check that we make is whether increases in housing wealth (that were perhaps unanticipated) over the period we consider could explain the greater growth in consumption among the more educated. We investigate this by running a median regression of the ‘ex-post’ discount rate on education dummies and a variable that gives the increase in housing wealth between the first and third waves of ELSA (2002 and 2006) as a proportion of initial wealth. The results are given in Table 9. Column (1) shows the results of a median regression on education dummies for our full sample. Column (2) shows the results of this regression applied to the 65 % of our sample who own their own property. Column (3) adds to this the increase in housing wealth as a proportion of total wealth. This coefficient is insignificant and the other coefficients barely change. We interpret this as evidence that changes in house prices and their effect on consumption do not explain our results.

**Table 9:** Discount rates and housing price growth

	(1)	(2)	(3)
Mid Education	1.33	−0.04	−0.06
High Education	9.62***	9.87**	9.87**
Housing wealth effect			−0.02
Constant	−3.16***	−3.41*	−3.42*
Observations	1,503	986	986

*** *p*<0.01, ** *p*<0.05, * *p*<0.1

As a final comment, we acknowledge the role that measurement error could play in our results. Note that classical measurement error, for example with a constant variance error that is multiplicative with consumption, will affect the level of the estimated discount rates but not affect the relative position of the groups. One needs a non-standard type of measurement error (for instance multiplicative with higher variance among those with more education relative to those with less) to undo our results.

## Conclusion

This paper puts forward a method for estimating individual discount rates using field data. We build consumption panel data from the intertemporal budget constraint and panel data on income and wealth. This household-level panel data is used, with the Euler equation, to estimate discount rates for groups defined by socio-economic characteristics.

We show, unsurprisingly, that there is substantial heterogeneity in discounting in our sample which is drawn from a population of older households. But surprisingly, we find that, among this older population, households with less education and lower numerical ability exhibit greater patience than, respectively, those with more education and greater numerical ability. This result, which is robust to differential housing wealth shocks, differential mortality and differential incidence of liquidity constraints is somewhat puzzling as it is the opposite to that found in investigations of time preference for younger households.

## Figures and Tables

**Fig. 5:**
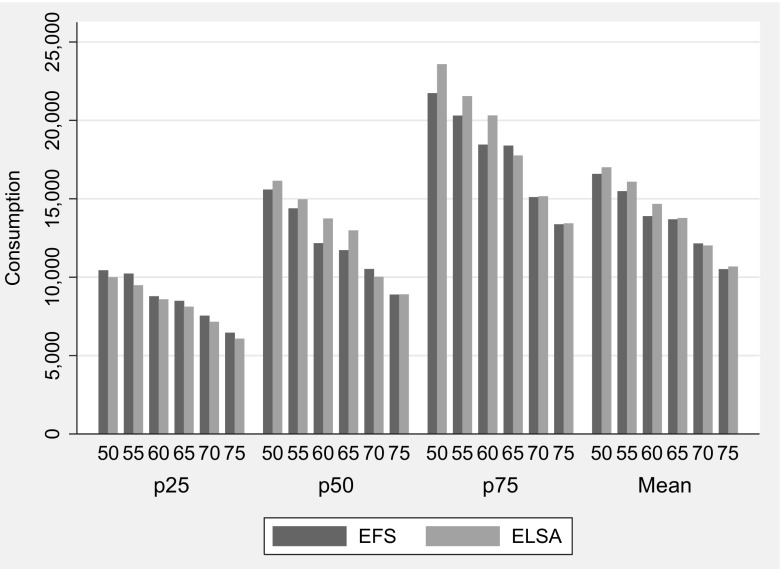
Comparing consumption in EFS and ELSA—by age—2003

**Fig. 6:**
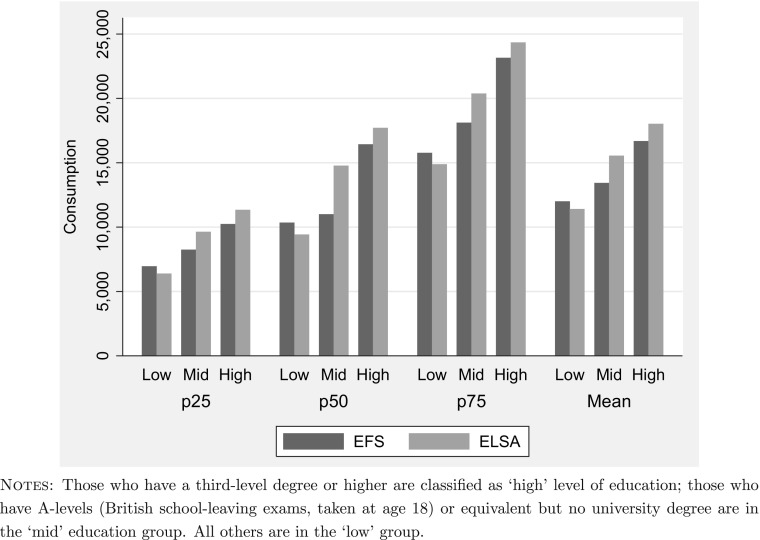
Comparing consumption in EFS and ELSA – by education – 2003

**Fig. 7:**
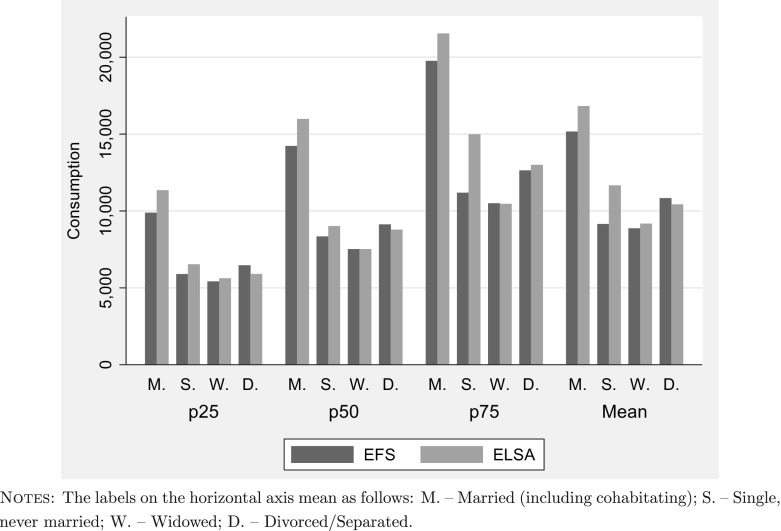
Comparing consumption in EFS and ELSA—by marital status—2003

**Fig. 8:**
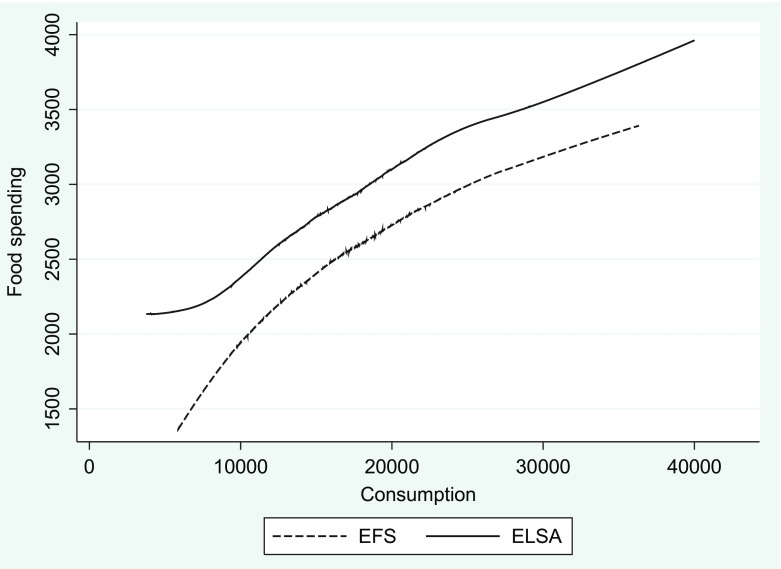
Comparison of relationship between food spending and consumption in EFS and ELSA—2003

## Appendix:

This appendix gives additional detail on the data that we use, our mr calculating consumption and the derivation of the sampling weights that we use. Section A presents statistics on the measures of wealth, education and numeracy in ELSA. Section B details the procedure by which we computed consumption from our panel data on wealth and income. Section C presents additional comparisons of our estimates of consumption from ELSA data with the consumption data available in EFS. Section D presents the characteristics of the final sample and, addressing concerns about our use of a non-random sub-sample of the population, discusses the construction of survey weights.

### A.1 Data

The data we utilise in this paper is the English Longitudinal Study of Ageing (ELSA). ELSA is a biennial longitudinal survey of a representative sample of the English household population aged 50 and over (plus their partners). The first wave was conducted between April 2002 and March 2003 and sampled 12,099 individuals (of whom 11,391 were core sample members; the remainder were individuals aged under 50 who were the partners of core sample members). There are 7894 benefit units (i.e. a single person or couple along with any dependent children) containing a sample member. Our sample is drawn from these benefit units.

ELSA collects a wide range of information on individuals’ circumstances. This includes detailed measures of their financial situation: income from all sources (including earnings, self-employment income, benefits and pensions), non-pension wealth (including the type and amount of financial assets, property, business assets and antiques) and private pension wealth (including information on past contributions and details of current scheme rules). ELSA also collects information on individuals’ physical and mental health, cognitive ability, social participation and expectations of future events (such as surviving to some older age or receiving an inheritance).

#### A.1.1 Data on household wealth

Given the importance of the wealth measure for our estimation, it is worth detailing how it is measured in the survey. ELSA respondents are asked for details on 16 different financial asset and (non-mortgage) debt instruments.^9^ For each asset (X), the ‘main respondent’ in each benefit unit is asked: ‘How much do you/you and your husband/wife/partner currently have in X?’. If the respondent does not know or refuses to say, a series of questions is asked that attempts to put lower and upper bounds on these assets. An imputation procedure is then carried out that gives a point estimate for the asset level for these individuals.^10^

Table 10 summaries the holdings in each of these assets. Some are self-explanatory (e.g. cash savings), others are specific to the UK and deserve some comment. Cash ISAs (Individual Savings Account) and TESSAs (Tax-Exempt Special Savings Account) are tax efficient cash savings which are subject to annual limits on what can be paid in. Stocks and shares ISAs and Personal Equity Plans (PEPs) are stocks and shares held in a tax-efficient ISA (Individual Savings Account) and are also subject to annual limits on what can be paid in. Life insurance savings ISA are life insurance savings held in a tax-efficient vehicle. Bonds could be either savings bonds (with retail banks) or government/corporate bonds. According to the ONS (2009) (Table 4.1) fewer than 2 % of households directly hold government or corporate bonds while over 8 % hold fixed-term bonds with financial institutions. Therefore, households in ELSA who report holdings of ‘bonds’ are more likely to be holding savings bonds (which are effectively fixed-term risk free savings accounts) than gilts or corporate bonds. National savings are cash savings held in the a government-owned agency (‘National Savings and Investment’). Finally, premium bonds are also issued by the government. Instead of yielding interest, the holders of these bonds are included in a monthly draw for large cash prizes.

**Table 10:** Holdings of different financial assets

Asset	(1)	(2)	(3)	(4)	(5)	(6)
	Mean	Mean	Med	Proportion	Mean	Prop.
	(Uncond.)	(Cond.)	(Cond.)	with asset	port. share	unimp.
Cash Savings	12,111	13,474	4000	90.1 %	54.9 %	80.4 %
Cash ISAs	2436	7452	6000	34.1 %	10.0 %	91.6 %
TESSAs	1457	9796	9000	16.7 %	3.5 %	95.3 %
National savings	832	11,547	3000	9.2 %	1.8 %	96.6 %
Premium bonds	763	2373	100	33.6 %	2.6 %	95.7 %
Bonds	2837	29,425	16,000	11.6 %	3.6 %	95.2 %
Shares	6650	22,087	3500	31.6 %	7.5 %	88.8 %
S&S ISAs	1551	11,982	7000	14.8 %	2.9 %	92.3 %
PEPs	2792	18,158	9000	17.2 %	3.7 %	93.1 %
Invest. trusts	2379	26,483	12,000	10.9 %	2.5 %	94.8 %
Life ins. savings	2267	22,470	10,000	12.0 %	4.5 %	93.2 %
Life ins. ISAs	91	9974	2000	3.0 %	0.2 %	95.4 %
Other savings	2179	40,458	15,000	7.4 %	2.4 %	96.9 %
Total	38,346	41,258	12,152	93.1 %	100.0 %	64.7 %

Notes: Column (1): mean holdings in the asset, *unconditional* on having a positive holding (in GBP); column (2): mean holdings in the asset, *conditional* on having a positive holding; column (3): median holdings in the asset, *conditional* on having a positive holding; column (4): the proportion benefit units that holds this asset; column (5): the mean portfolio share (among those with positive total gross assets); column (6): the proportion of benefit units who report their exact holdings and so for whom no imputation is necessary

Sources: ELSA, wave 1 (2002/03)

The ELSA survey asks also for details on three different types of non-mortgage debt. These are credit card debt, private debt (i.e. debts to friends and family) and ‘other debt’ (primarily overdrafts and personal loans). Table 11 summarises the holdings of different debt instruments and has a form similar to Table 10.

**Table 11:** Balances of different debts

Debt type	(1)	(2)	(3)	(4)	(5)	(6)
	Mean	Mean	Med	Proportion	Mean	Prop.
	(Uncond.)	(Cond.)	(Cond.)	with ass.	port. share	uinimp.
Credit card debt	369	1,989	800	20.3 %	41.1 %	98.8 %
Private debt	68	5,612	1,000	3.3 %	2.8 %	99.3 %
Other debt	846	3,904	1,500	23.3 %	56.1 %	98.9 %
Total	1,293	4,067	1,400	31.8 %	100.0 %	97.1 %

Notes: See notes to Table 10

Sources: ELSA, wave 1 (2002/03)

#### A.1.2 Data on education and numerical ability

We outline here how our measures of education and numerical ability are defined.

We categorise individuals into one of three education groups on the basis of the highest qualification that they have. We consider those who have a third-level degree or higher to have a ‘high’ level of education. Those who have A-levels (British school-leaving exams, taken at age 18) or equivalent but no university degree are in the ‘mid’ education group. All others are in the ‘low’ group. Our measure of consumption, and therefore our estimates of discount rates, are at the household (formally benefit unit^11^) level. We need an education measure, therefore, at the household level. We take the education of a household containing a couple to be the greater of the two levels of education held by the adults in that couple.

Numerical ability is measured in ELSA using a series of six questions, which are reproduced in Box 1. The simplest of these questions requires the respondent to solve a very simple exercise in subtraction while the most difficult requires the respondent to solve a problem involving compound interest. We divide all individuals into one of four groups following the categorisation in Banks et al. (2010). Mirroring our approach with respect to education, we take the numerical ability of a household containing a couple to be the greater of the two levels of numerical ability held by the two members of that couple.

### A.2 Computing consumption

In this subsection, we present some additional details on the consumption calculation procedure. We reproduce Eq. 8 here, which makes clear the data requirements:

10 $$ p_{t} c_{t} = e_{t} + t_{t} + \sum\limits_{j} \left( {q^{j}_{t}} + \frac{ p^{j}_{t+1} - {p^{j}_{t}}}{ {p^{j}_{t}}}\right) {p^{j}_{t}} {X^{j}_{t}} + \sum\limits_{j} \left( {p^{j}_{t}} {X^{j}_{t}} - p^{j}_{t+1} X^{j}_{t+1}\right)  $$

Three issues that we now discuss in turn are the following:

1. How to estimate capital gains on assets held between the two waves $\left (\frac { p^{j}_{t+1} - {p^{j}_{t}}}{ {p^{j}_{t}}}\right )$
2. How to estimate income and transfers for the first half of the period between waves. This is necessary as ELSA sample members are surveyed approximately every two years with the questionnaire seeking, in most cases, data on receipts in the past year, rather than on over the entire period between waves
3. Whether to make adjustments to the procedure when asset stocks in either wave are imputed

#### A.2.1 Estimating capital gains

We need to estimate capital gains on assets held between two waves $\left (\frac { p^{j}_{t+1} - {p^{j}_{t}}}{ {p^{j}_{t}}}\right )$. Depending on the type of asset we make different assumptions.

i. For cash or most cash-like assets (savings, TESSAs, National savings, Prize bonds) and ‘other savings’, we assume no capital gain $\left (\frac { p^{j}_{t+1} - {p^{j}_{t}}}{ {p^{j}_{t}}} = 0\right )$.
ii. For Cash ISAs and Bonds,^12^ we have a concern that some respondents, for whom their interest income is simply being rolled up in their account and not withdrawn, will report that their ‘income’ is zero, when in fact it is positive but simply saved. If income from the asset is reported as positive, we assume that there is no change in the value of the asset. If, however, individuals report zero income from their holdings (and approximately 39 % of bondholders do in wave 1), we assume that they *are*, in fact, receiving interest and that this interest is simply accumulating in their account and they don’t as a result consider it as ‘income’. We assume that they earn a rate of return equal to the median rate of return for those holding similar assets who *do* report income and reflect this interest in an increase in the ‘value’ of the asset. That is, for individuals who hold the particular asset but who report no income, we assume that, if the median return on that asset in that wave was 2 % then $\frac {p^{j}_{t+1} - {p^{j}_{t}}}{ {p^{j}_{t}}} = (1.02)^{\tau } - 1$, where *τ*≈2 is the length of time in years between the two interviews.
iii. For equities and equity-like assets, we make a distinction whether the income from the asset is reported as positive or zero. If income is reported as positive, we assume that the value of the asset increased in line with the FTSE 100 price index (which excludes dividend payments) between the dates of the two interviews. If income is reported as zero, we assume that the value of the asset increased in line with the FTSE 100 *total return* index (i.e including dividend payments) between the dates of the two interviews.
iv. For debt, we assume that the interest rate on credit card debt is 15 %, the interest rate on ‘other debt’ (mostly overdrafts) is 8 % and that there is a 0 % interest rate on ‘private debt’.

#### A.2.2 Imputing missing income

For each month between the waves where we do not have income data, we interpolate linearly between the two income observations that we have. We carry out this procedure separately for each category of income (employment income, self-employment income, private pension income, state pension income, benefit income, asset income and other income).

We vary the procedure in two cases. The first of these is when respondents do not report some category of their income exactly in one (but not both) of the waves (i.e. they perhaps only give bounds). In these cases we assume that income has been equal over the period to the value in the year for which we have full information. The second case where we vary the procedure is when it comes to state pension income. Here we use the data on the age of the respondents and the state pension age to establish when their state pension payments started.

#### A.2.3 Whether to make adjustments to the procedure when asset stocks in either wave are imputed

In a number of cases, survey respondents do not know the exact amount of some asset holding or some income component. In that case, the ELSA questionnaire attempts to obtain bounds on the unknown amounts and the survey data is published with imputed amounts that lie between these bounds.

When individuals report that they do not know exactly their holdings in any particular wave *t* or *t*+1 (i.e. they don’t know ${X^{j}_{t}}$ or $X^{j}_{t+1}$ for some *j*), we make the assumption that they made no payments into or withdrawals from their assets—i.e. ${X^{j}_{t}} - X^{j}_{t+1} = 0$.

### A.3 Comparisons of consumption measures between EFS and ELSA

Figures 5, 6 and 7 probe the comparison between the distribution of consumption in the EFS and in ELSA more deeply than in the body of the paper. The figures each take a particular household characteristic (age, education and marital status respectively) and compare the conditional distributions of consumption in both surveys. We show the 25th, 50th and 75th percentiles as well as the mean. Of interest are both the difference between quantiles for a households of a particular type (for example, whether the shape and location of the distributions match for young people) and the differences for a particular quantile across household types (for example, whether the relationship between median expenditure and age is similar in both surveys).

These figures show that many of the points we made above in our comparison of the unconditional distributions of consumption between surveys are true for the distributions conditional on (at least these) household characteristics. For all age groups, for those with middle and higher levels of education and for all marital statuses, the 75th percentile of consumption is higher in ELSA than in the EFS, and in most cases the median and mean are higher too, with smaller differences to be seen between the 25th percentiles in each survey. The socio-economic gradients observed in the EFS are closely replicated in the ELSA consumption data – that is consumption decreases with age, rises with education and married households consume more than single households (unsurprisingly as our measure of consumption here is not adjusted using an equivalence scale).

As a final comparison of our generated data on consumption with that in the EFS we make use of the fact that data on food spending is recorded in ELSA. In Fig. 8, we plot the relationship between food spending and total spending in both surveys (again trimming the bottom and top 10 % of consumption). The relationships shown are estimated using locally-weighted regressions. Food spending is estimated as greater in ELSA than in the EFS;^13^ the slope of the relationship between it and total spending is, however, similar in both surveys from the point at which total consumption is equal to approximately £10,000. Below this level, food spending in ELSA does not vary much with our calculated measure of total consumption—an indication, perhaps, that some of the households in the left-tail of the distribution of consumption are there due to measurement error.

### A.4 Selection and composition of sub-sample

To balance considerations of maintaining as large a sample as is possible and of using as little inaccurate data as is possible, we exclude observations where we feel the data did not allow us to estimate consumption. We provide here details on how our sample was selected (D.1) and then we describe the selected sample and the construction of weights used to correct for our use of a non-representative sample in (D.2).

#### A.4.1 Selection of the sample

We exclude benefit units from the our estimating sub-sample if any of the following conditions hold.

1. If at least one component of income is not known up to a closed interval in *both* waves *t* and *t*+1.
2. If a ‘large’ change in physical wealth was observed between the two waves. Changes in the financial asset values that we observe could then be the result of transferring asset holdings between types of assets rather than being indicative of consumption. We define a large change in ‘physical assets’ as occurring when there has *both* been a change in the value of holdings of at least £2,000 *and* a proportionate change in the value of the portfolio of at least 30 %
3. If a ‘large’ change in uncategorised wealth was observed between the two waves. A small number of couples keep their finances largely separate. When these individuals are asked for any asset holdings that they hold jointly, only the total level of jointly-held asset is sought - not the level of holdings of particular assets. Estimating the capital gain between waves is thus not possible. A large change is the value of joint holdings is considered to have occurred when there has *both* been a change in the value of holdings of at least £2,000 *and* a proportionate change in the portfolio of at least 30 %
4. If an individual bought or sold a house between the waves
5. If mortgage payments are missing
6. If some critical piece of data is missing (usually education or age), the imputation procedure used by the ELSA team to fill in missing income and asset holdings is not possible
7. If the benefit unit composition changed between the two waves
8. If the either partner in a couple does not respond to the survey
9. If a lump-sum payment has been received in the past year and we do not observe the exact amount
10. If either member of a benefit unit suffered the bereavement of their last remaining living parent between waves (and thus may have received an inheritance that we might not observe if was received over a year before the ELSA interview)

Table 12 summarises the selection operated by our procedure from the initial sample. We need two successive observations on assets to compute consumption – therefore the left panel shows the proportions for whom we have a successful consumption calculation as a proportion of the 6,022 benefit units who make up the balanced panel for waves 1 and 2 (the panel on the right of the table shows these proportions out of all those observed in wave 1. The rules we have established above lead us to exclude 38 % of the balanced panel. For an additional 2.3 % of the sample, we estimate negative consumption level, indicative of a failure to impute consumption.

**Table 12:** Success rate of consumption calculation

Computation status	Proportions of balanced panel		Proportions of wave 1 sample	
	Obs.	Percentage	Obs.	Percentage
Have consumption	3,541	58.8 %	3,541	44.8 %
Calculation failed	2,298	38.2 %	2,298	29.1 %
Negative consumption	183	3.0 %	183	2.3 %
Attrited	–	–	1,872	23.7 %
Total	6,022	100.0	7,894	100.0 %

Table 13 summarises the proportions of benefit units in the balanced panel where a particular reason for the consumption calculation being unsuccessful is relevant.

**Table 13:** Summary of reasons for consumption calculation failing

Reasons	Percentage	
Missing income component	760	12.6
Large change in physical wealth	721	12.0
Large change in uncategorised wealth	85	1.4
Bought or sold property	321	5.3
Mortgage payments missing	48	0.8
Critical information missing	181	3.0
Benefit Unit composition changed	145	2.4
Non-responding partner	416	6.9
Missing data on lump-sum receipt	141	2.3
Last surviving parent died	352	5.9

We described above how we deal with cases where some component of assets or income is not known exactly and have had to be imputed. Where assets are not known in either wave, we assume that there has been no flow in or out between waves. We thus do not use the imputed data on assets. We do, on the other hand, use the imputed data on income, but exclude benefit units where at least one component of income is not known up to a closed interval in *both* waves *t* and *t*+1. We have assessed if (and confirmed that) our results hold if we apply stricter sample selection rules with respect to the use of imputed data. The third and fourth panels in Table 6 for example, show the estimated discount rates with successively stricter sample selection rules than applied in the baseline (in the text of the paper we have referred to these as our ‘middle’ and ‘strict’ sample selection rules). These rules are as follows:

- **Middle**: Do not include individuals when the value of some asset holding in wave *t* is not known at least up to a closed interval. Do not include individuals where at least one component of income is not reported exactly in *both* waves *t* and *t*+1.
- **Strict**: Do not include individuals when the value of some asset holding in wave *t* is not reported exactly. Do not include individuals where at least one component of income in wave *t* is not reported exactly in *either* wave *t* or *t*+1.

The foregoing tables and discussion detail the extent to which we have been able to calculate consumption. To estimate a discount rate we need, of course, at least two successive observations on consumption (and therefore three successive observations in the data). There are 4,886 benefit units in the balanced panel of the first three waves of data. Of these we can include 1,578 (32 %) of these in our estimating sub-sample. We exclude benefit units where the consumption calculation failed between either pair of waves (1 and 2 or 2 and 3), where calculated consumption is less than £3,000 in either case or where the benefit unit composition or labour supply activity of either member changed. Table 14 shows the number (and proportion) of cases in which these conditions are relevant.

**Table 14:** Summary of reasons for consumption calculation failing

Reasons	Percentage	
Consumption calculation failed between either pair of waves	2,532	51.8
Consumption less than £3000 between either pair of waves	780	16.0
Benefit unit composition changed	1,103	22.6
Labour supply changed	690	14.1

#### A.4.2 Characteristics of the selected sample

With a successful calculation of consumption for approximately 60 % of the balanced (wave 1 and 2) panel, and the inclusion in the estimating sub-sample of approximately 32 % of the balanced (waves 1 to 3) panel, there might be some concern about the representativeness of the sample. In this section we describe the dimensions along which these samples are non-random.

Table 15 contains a multivariate analysis of the probability of our having successfully calculated consumption (the left hand panel) and the probability of being in our discount rate estimating sub-sample (the right hand panel). Each panel contains the results from the two logistic regressions—the first where the sample is all those benefit units observed in wave 1, the second where the sample is the relevant balanced panel.

**Table 15:** Logit regression of successfully calculating consumption on sample on characteristics

	Probability of being in		Probability of being in	
	consumption sample		discount rate sample	
	(1)	(2)	(3)	(4)
Age 50 to 54 (omm.)				
Age 55 to 59	0.98	1.12	0.94	0.99
Age 60 to 64	1.00	1.11	0.99	1.07
Age 65 to 69	1.15	1.31***	1.77***	2.07***
Age 70 to 74	1.30***	1.69***	1.95***	2.56***
Age 75 to 79	1.21*	1.88***	1.66***	2.56***
Age 80 to 84	0.95	1.70***	1.35**	2.48***
Age 85 or over	0.59***	1.82***	0.76	2.44***
Single (omm.)				
Married	0.81**	0.77**	0.77**	0.83
Widowed	1.30***	1.12	1.10	0.88
Separated	1.29**	1.24	1.33**	1.29
Low Educ. (omm.)				
Mid. Educ.	1.15**	0.98	1.14	0.94
High. Educ.	1.03	0.89	0.96	0.80*
Inc. quint. 1 (omm.)				
Inc. quint. 2	1.01	0.94	0.94	1.00
Inc. quint. 3	1.09	0.97	0.90	0.83*
Inc. quint. 4	1.05	1.00	0.87	0.86
Inc. quint. 5	1.01	0.92	0.66***	0.61***
Wealth quint. 1 (omm.)				
Wealth quint. 2	0.83**	0.45***	0.71***	0.49***
Wealth quint. 3	0.72***	0.33***	0.57***	0.33***
Wealth quint. 4	0.56***	0.22***	0.41***	0.22***
Wealth quint. 5	0.35***	0.13***	0.21***	0.11***
Observations	7,253	5,476	7,253	4,439

Notes: In specifications (1) and (2) the sample is all those sampled in wave 1, regardless of whether they attrited by wave 2 or not. In specification (3) the sample is the balanced (wave 1 and 2) panel. In specification (4), the sample is the balanced (waves 1, 2 and 3) panel

*** *p*<0.01, ** *p*<0.05, * *p*<0.1

The dependent variables include dummies for age, marital status, education, quintiles of equivilised income and quintiles of wealth (odd ratios are presented). For couple, age is taken as the age of the male. The only other variable included in the analysis which requires some explanation is education. We categorise individuals as having one of three levels of education: low, middle and high. Those in the ‘low’ group either have no formal academic or vocational qualifications or have a Certificate of Secondary Education, those in the ‘middle’ group have A-levels or O-Levels and those in the ‘high’ group have a higher-level degree. Some individuals have qualifications which don’t fit neatly into one of these categories (because for example, they have foreign qualifications or have some higher level education below degree). We place these individuals in the low, middle and high education group respectively if they left full-time education at or before the age of 16, at 17 or 18, or at or later than 19.

The sub-samples that we use are indeed non-random samples from the entire ELSA sample—with an under-representation of younger benefit units, married benefit units and benefit units with more education. To account for this, we weight the observations in each empirical exercise. The weights are equal the product of the ELSA survey weights and the inverse of the probability of being included in the estimating subsample. This probability is estimated using the logistic regressions on the entire wave 1 subsample.

## Footnotes

1 Alternatively, we could specify a liquidity constraint that ensures that total debts are no greater than a certain quantity.

2 A notable exception to this is the Spanish ‘Encuesta continua de presupuesto familiares’, a diary-based longitudinal survey of expenditure. The Panel Study of Income and Dynamics in the US, since 1999, has collected expenditure data that covers approximately 70 % of average total expenditure.

3 There are also questions on housing wealth, physical wealth and pension wealth.

4 This survey has, since 2008, been known as the Living Costs and Food Survey. However, the data that we show is from years prior to this, so we make use of the older name.

5 Those who did not complete UK school leaving exams (typically taken at the age of 18) have ‘low’ education, those who completed these but have no third-level education are in the ‘middle’ education group, and those with any post-schoolqualification are in the ‘high’ education group. Numerical ability is tested using six questions in ELSA—our categorisation follows that in Banks et al. (2010). Marital status is defined covering the first four waves of ELSA - those whose status changed are in the ‘other’ group. The age, education and numerical ability of the couple are taken as that of the older/more educated/more numerically-able of the couples. More details on the education and numerical ability characteristics are given in Appendix A.

6 These results are not directly comparable with those in Alan and Browning (2010) where the distributions are presented graphically. Additionally, that paper discusses how their results relate to those in Samwick (1998). Alan and Browning (2010) restrict the discount rate to be greater than 0. We find, as do GS and Samwick, evidence of households with negative discount rates.

7 While we refer to (the absence of) a relationship with age, we are not able to separately identify associations of discount rates with each of age and cohort. Exploiting a longer panel than we have access to would allow us to separately identify age and cohort effects if we assumed that there were no time effects in discount rates.

8 The differences are also statistically significant. The difference between the mean discount rates of the low and middle education groups are significant at the 5 % level and those between the high and low education group are significant at the 1 % level.
